# Adaptive Immunodeficiency in WHIM Syndrome

**DOI:** 10.3390/ijms20010003

**Published:** 2018-12-20

**Authors:** Shamik Majumdar, Philip M. Murphy

**Affiliations:** Molecular Signaling Section, Laboratory of Molecular Immunology, National Institute of Allergy and Infectious Diseases, NIH, Bethesda, MD 20892, USA; shamik.majumdar@nih.gov

**Keywords:** B lymphocytes, T lymphocytes, chemokines, CXCL12, NK cells, human papillomavirus, HPV

## Abstract

Cysteine-X-cysteine chemokine receptor 4 (CXCR4) is a broadly expressed and multifunctional G protein-coupled chemokine receptor critical for organogenesis, hematopoiesis, and antimicrobial host defense. In the hematopoietic system, the binding of CXCR4 to its cognate chemokine ligand, CXCL12, mediates leukocyte trafficking, distribution, survival, activation, and proliferation. Warts, hypogammaglobulinemia, infections, and myelokathexis (WHIM) syndrome is a rare, autosomal dominant, combined immunodeficiency disorder caused by mutations in the *C*-terminus of CXCR4 that prevent receptor downregulation and therefore result in pathologically increased signaling. The “M” in the acronym WHIM refers to myelokathexis, the retention of neutrophils in the bone marrow resulting in neutropenia, which explains in part the increased susceptibility to bacterial infection. However, WHIM patients also present with B and T lymphopenia, which may explain the susceptibility to human papillomavirus (HPV), the cause of warts. The impact of WHIM mutations on lymphocytes and adaptive immunity has received less attention than myelokathexis and is the focus of this review.

## 1. Introduction

**Figure 1:**
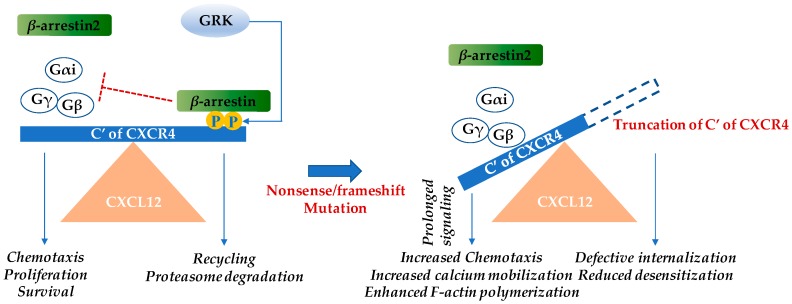
Warts, hypogammaglobulinemia, infections, and myelokathexis (WHIM) mutations in the cytoplasmic region of the cysteine-X-cysteine chemokine receptor CXCR4 result in gain-of-function. Upon CXCL12 binding, CXCR4 undergoes conformational changes and the intracellular trimeric G-protein is activated. The Gα subunit dissociates from the βγ-subunit and various pathways including the Ras/Raf/ERK (extracellular regulated kinase), PI3K (phosphoinositide 3-kinase), PLC (phospholipase C), PKC (protein kinase C), and mitogen associated protein kinase (MAPK) pathways are activated. These subsequently result in chemotaxis, increased proliferation, and cell survival [11]. *β*-arrestin 2 also augments chemotaxis by enhancing p38 MAPK activation [9,12]. Inhibition of CXCR4 signaling occurs by the *β*-arrestin pathway. CXCL12 binding recruits GPCR kinase (GRK), which induces site-specific phosphorylation at the *C*-terminus of CXCR4. *β*-arrestin associates with CXCR4 and triggers receptor internalization. Subsequently, either the chemokine receptor is recycled back to the cell surface or it is degraded by the proteasome. In WHIM syndrome, most mutations cause truncation of the *C*-terminus of CXCR4 and the phosphorylation sites of GRK for *β*-arrestin docking are no longer available, leading to reduced desensitization and prolonged signaling [8,10]. The blue arrows indicate induction, while the red “T” bar denotes inhibition.

Warts, hypogammaglobulinemia, infections, and myelokathexis (WHIM) syndrome (OMIM 193670) is an extremely rare, combined primary immunodeficiency disorder found worldwide, that is estimated to have an incidence of 0.23 cases per million births [1]. According to the Orphanet Report Series-Prevalence of rare diseases: Bibliographic data, as of June 2018 only 65 cases of WHIM syndrome had been reported in the literature [2]. The age at diagnosis and clinical presentation may be highly heterogeneous [1,3,4,5,6]. Almost all WHIM patients have myelokathexis and recurrent infections, and most but not all will eventually develop refractory warts [5]. Hypogammaglobulinemia is the least penetrant phenotype. Myelokathexis refers to retention of neutrophils in the bone marrow [7]. All but a few cases of WHIM syndrome are caused by autosomal dominant gain-of-function mutations in the G protein-coupled, cysteine-X-cysteine chemokine receptor CXCR4 [8], and these are the only chemokine or chemokine receptor mutations responsible for a Mendelian condition. Heterozygous nonsense or frameshift mutations result in truncations of the cytoplasmic tail domain of the receptor, which is rich in serine and threonine residues. Upon receptor activation, this domain normally becomes phosphorylated by G protein–coupled receptor kinases and binds *β*-arrestin which mediates receptor internalization/downregulation. Therefore, the deletion of portions of the *C*-terminal domain of CXCR4 prolongs receptor residence time on the cell surface and amplifies receptor signaling [9] (Figure 1). One cellular consequence of this is that cells expressing the WHIM variant of CXCR4 demonstrate greater chemotaxis in vitro towards CXCL12 (previously known as stromal cell-derived factor-1α) [10]. 

Myelokathexis is key to the clinical diagnosis of WHIM syndrome. There are only two other diseases in which a version of this condition has been described, G6PC3 deficiency and GATA2 deficiency, both of which are usually easily distinguished from WHIM syndrome by other differential clinical manifestations. The term myelokathexis was coined by Zuelzer et al. in 1964 to describe severe congenital neutropenia despite complete maturation of myeloid cells in the bone marrow in a 9-year old girl who presented with recurrent bacterial infections [5], the first report of a “WHIM-like syndrome” in the literature [13,14]. She is also the first WHIM patient to be cured naturally of her disease, by chromothriptic deletion of the WHIM allele on chromosome 2 in a single hematopoietic stem cell (HSC) that fortuitously acquired a growth advantage and repopulated her bone marrow. This patient is now followed at the National Institutes of Health (NIH) and has been designated as WHIM-09 [15]. 

Chromothripsis, which is a neologism meaning “chromosome shattering”, was first described in the cancer literature. One or several chromosomes shatter and a subset of the fragments rejoin in a random order to form a derivative chromosome marked by multiple genomic deletions and sometimes duplications. In the case of WHIM-09, this appears to have happened as an adult and on one copy of chromosome 2, resulting in the loss of one copy each of 164 genes, one of which was the WHIM allele of *CXCR4*. Interestingly, although her HSCs and her entire myeloid lineage appear to be ~100% chromothriptic and non-WHIM, her entire lymphoid lineage remains WHIM and non-chromothriptic. Thus, she continues to have circulating B and T lymphopenia as well as hypogammaglobulinemia, but has mild neutrophilia and monocytosis, not neutropenia or monocytopenia. Since she no longer has warts or experiences recurrent bacterial infections, silencing of the WHIM allele in the myeloid lineage appears to have been sufficient to abrogate the major clinical manifestations of the disease [15]. *Cxcr4* hemizygosity per se enhances HSC proliferation and engraftment in the context of transplantation in congenic mice, suggesting a specific mechanism by which the initial chromothriptic HSC might have attained dominance and providing a clinical cure mechanism for WHIM-09 [16].

Neutropenia may change to neutrophilia when WHIM patients become infected, which may serve to attenuate the severity of WHIM infections and explain why invasive and life-threatening infections are uncommon in the disease [14,17,18,19]. Since WHIM syndrome is a subtype of severe congenital neutropenia (SCN), patients are commonly treated with granulocyte colony-stimulating factor (G-CSF/filgrastim, Neupogen; Amgen Inc., Thousand Oaks, CA, USA), although its safety and efficacy in WHIM syndrome have never been established in clinical trials. Immunoglobulin (Ig) supplementation and prophylactic antibiotics are also administered to counter hypogammaglobulinemia and infections, but also have not been evaluated in clinical trials [7,20]. For human papillomavirus (HPV)-induced lesions, laser ablation, surgical excision, and cryotherapy are used in WHIM patients, whereas pharmacologic agents such as imiquimod have not been reported to be effective in the disease [20]. 

In mice, complete *Cxcr4* deficiency results in congenital defects in cardiac ventricular septum formation, central nervous system development [21,22], vascularization of the gastrointestinal tract [23], and hematopoiesis [22,24], as well as perinatal mortality. Congenital cardiovascular defects, including tetralogy of Fallot, have also been observed in some patients with WHIM syndrome [1,4,17,20,25]. Together, these phenotypes have cautioned clinical trials of CXCR4-targeted therapy. Nevertheless, progress has been made (see below). 

Although WHIM syndrome is characterized by myelokathexis, most patients have panleukopenia, including lymphopenia, associated with hypogammaglobulinemia. Defects in somatic hypermutation and isotype switching of immunoglobulin loci as well as poor responses towards vaccinations have been reported in some patients [1]. CXCR4 is highly expressed on most subsets of lymphocytes, and its cognate ligand, CXCL12, is broadly expressed. In the immune system, it is especially highly expressed in primary and secondary lymphoid organs, consistent with diverse roles for CXCR4 in lymphocyte development, trafficking, and activation. Unlike other reviews of WHIM syndrome, the remainder of this review will focus on CXCR4 and WHIM mutations in adaptive immunity in patients and in the mouse model of WHIM syndrome [26].

## 2. Lymphoid Organs

The bone marrow and thymus constitute the primary lymphoid organs where B and T lymphocytes develop and mature. In secondary lymphoid organs, such as lymph node, spleen, Peyer’s patches, and mucosa-associated lymphoid tissues, lymphocytes survey tissue for antigen and mount adaptive immune responses upon antigen encounter.

### 2.1. Bone Marrow

HSCs in the bone marrow occupy distinct perivascular niches and give rise to cells of all the hematopoietic lineages. Early lymphoid progenitors occupy an endosteal niche [27]. The highest expression of CXCL12 is found in the bone marrow (https://www.proteinatlas.org/ENSG00000107562-CXCL12/tissue#gene_information, last accessed on 18 December 2018), where it is mainly produced by endothelial cells and perivascular stromal cells. HSCs contact CXCL12-abundant reticular cells, which are adjacent to sinusoidal endothelial cells and endosteum [28]. Hematopoiesis is extremely sensitive to the strength of CXCL12-CXCR4 signaling, which is required for maintenance [28,29] and quiescence [30] of HSCs as well as long-term reconstitution of myeloid and lymphoid cells [31]. The bone marrow of both *Cxcl12*- and *Cxcr4*-deficient embryos is hypocellular and is mainly composed of stromal cells; osteoblasts and hematopoietic cells of all lineages are significantly reduced [32]. Conversely, increasing CXCR4 function in the bone marrow, as assessed using the *Cxcr4^+/1013^* WHIM mouse model, reduces bone marrow content of lymphoid-primed multipotent progenitors (LMPPs, Lin^−^c-Kit^+^Sca-1^+^ [LSK] Flt3^high^CD34^+^) and CLPs (Lin^−^c-Kit^low^Sca-1^low^Flt3^+^CD127^+^), whereas the numbers of erythroid and myeloid progenitor cells remain intact [33]. Hemizygous *Cxcr4*^+/o^ mice have normal bone marrow architecture and HSC and progenitor cell distribution; however, as mentioned previously, HSCs from these mice have superior long-term bone marrow engraftment capacity after transplantation in both lethally irradiated and non-conditioned congenic recipients compared to *Cxcr4^+/1013^* or *Cxcr4^+/+^* HSCs [34]. The mechanism appears to involve increased HSC proliferation and enhanced mature leukocyte release to the blood [15,34].

Bone marrow biopsies from WHIM patients are hypercellular [1,14,17,18,25,35,36,37,38] with elevated proportions of mature granulocytes and lymphocytes and an elevated myeloid to erythroid ratio [39]. Many neutrophils appear to be apoptotic with hypersegmented hyperdense nuclei, wispy strands connecting the nuclear lobes, and cytoplasmic vacuoles [5,17,36]. The small molecule CXCR4-specific inhibitor AnorMED3100 (AMD3100), also known as plerixafor and Mozobil^®^ (Sanofi, Paris, France) [40], has been used in clinical trials to relieve neutropenia in WHIM patients by mobilizing the large pool of non-circulating neutrophils. AMD3100 was initially developed as an HIV entry inhibitor. It failed that indication because most strains of HIV in patients use CCR5 for entry and because of arrhythmias noted in some patients during clinical trials. The drug was then repurposed for HSC mobilization and is currently US Food and Drug Administration-approved for use in combination with G-CSF in patients with non-Hodgkin’s lymphoma and multiple myeloma to mobilize and collect HSCs for autologous bone marrow transplantation after chemotherapy [41,42]. In both healthy individuals and WHIM patients, AMD3100 also induces panleukocytosis in a dose-dependent manner [38,43,44]. A phase 1 clinical trial of long-term, low-dose AMD3100 administration in three WHIM patients conducted at the NIH demonstrated mobilization of leukocytes to blood and reduced hypercellularity of the bone marrow. In particular, the frequencies of neutrophil and CD19^+^ B cells in the bone marrow were reduced, whereas monocyte frequencies increased. In two of the three patients, the frequency of CD3^+^ T cells also increased [38]. No serious adverse events were noted in this limited study, and wart burden and infection frequency were noted to decrease.

### 2.2. Thymus 

T cell development, selection, and maturation occur in the thymus. The progenitor T cells arrive from the bone marrow to the thymus and undergo sequential development. Unlike inflammatory chemokines which are produced under conditions of stress, CXCL12 is a constitutive chemokine expressed in the thymus under homeostatic conditions. In adult mice, Cxcr4 contributes to homing of thymic progenitors to the thymus from the bone marrow [45]; however, it is not indispensable [46]. Thymocytes can be broadly classified into four developmental stages based on T-cell coreceptor expression as CD4^−^CD8^−^ (double negative, DN), CD4^+^CD8^+^ (double positive, DP), CD4^+^CD8^−^ (single positive [SP] CD4), and CD4^-^CD8^+^ (SP CD8). 

In human thymus, CXCL12 is produced by epithelial cells localized in the subcapsular and medullary regions. Surface expression of CXCR4 is upregulated by IL-7 stimulation of CD34^+^ progenitor cells committed to the T-cell lineage, which in turn enhances their survival and proliferation [47]. During development, thymocytes downregulate cell surface CXCR4 expression. Accordingly, calcium flux responses to CXCL12 stimulation are detected in DN and DP but not CD4 SP thymocytes [48]. In the mouse thymus the most immature thymocytes, in the DN subset, are conventionally phenotypically identified as DN1 (CD4^-^CD8^−^CD44^+^CD25^−^), DN2 (CD4^−^CD8^−^CD44^+^CD25^+^), DN3 (CD4^−^CD8^−^CD44^−^CD25^+^), and DN4 (CD4^−^CD8^−^CD44^−^CD25^−^). Immature DN2 and DN3 thymocytes express the highest levels of CXCR4 transcripts, and cortical but not medullary stromal cells express CXCL12. CXCR4 and CXCL12 interaction drives the early immature thymocytes from the site of entry of T cell progenitors in the thymus, that is, the corticomedullary junction (CMJ) to the cortex for further development. Not surprisingly, in the postnatal thymus, thymocytes lacking CXCR4 are found at the CMJ and reportedly undergo developmental arrest at the DN1 stage [49]. However, a subsequent study refuted this observation stating that CXCR4 is required at the DN3-DN4 stage rather than at the DN1-DN2 stage of development [50]. CXCR4 signaling activates PI3K in DN3 thymocytes and facilitates Notch-dependent development of these cells into DP thymocytes [51]. In addition, CXCL12–CXCR4 interaction also provides costimulatory signals and facilitates β-selection during which the DN cells carrying a productively rearranged TCR β-chain undergo expansion and progression [50]. In this way, the cellularity of the DN3, DN4, and DP cells in fetal thymi of mice is increased [52]. Contradictory results were presented in another study wherein CXCR4 was dispensable in the embryonic thymi. The *Cxcr4*-deficient embryos demonstrated normal thymic cellularity and cellular distribution, and the DN cells developed to become SP cells in in vitro fetal thymic organ culture [32]. The authors attributed this to the redundant roles of CCR7, CCR9, and CXCR4 in homing of thymocytes before and after thymus vascularization. In addition, thymi obtained from mouse embryos lacking all three chemokine receptors were deficient in early thymic progenitors, which are phenotypically characterized as Lin^-^CD117^+^CD25^−^ [46].

The role of CXCL12–CXCR4 interaction in T cell development was thought to be limited to the DN stage, as its deletion at the DP and SP stages did not affect the selection and maturation of thymocytes [53]. The thymus contains a CXCR4^hi^ population of DP thymocytes which are large cycling cells expressing high amounts of the major glucose transporter, glucose transporter 1 (GLUT1) and the transferrin receptor CD71 that have undergone β-selection [54]. However, the physiological significance of these cells is still unknown. After positive selection, when the thymocytes are CD69^+^CD3^+^ and CD69^−^CD3^+^, CXCR4 is downmodulated [54,55] most likely to assist in emigration of SP thymocytes from the thymus by fugetactic responses towards CXCL12 [56,57]. Furthermore, thymic dendritic cells also express CXCR4 and CXCL12 which enhance survival by upregulating the ratio of the survival factors Bcl2 and Bax [58]. In summary, CXCR4 is involved at multiple stages of T cell development, selection, and emigration from the thymus.

CXCR4, CCR5, and other chemokine receptors can be used selectively by HIV strains with CD4 as cell entry coreceptors. CXCR4-tropic HIV strains, in addition to infecting circulating CD4^+^ T cells using CXCR4 as the entry coreceptor [59,60], also infect CXCR4-expressing thymocytes. In vitro HIV preferentially infects and depletes immature thymocytes expressing the highest amounts of both CXCR4 and CD4 [61]. HIV is known to interact with thymic epithelial cells to induce IL-7 secretion, which in turn upregulates CXCR4 expression on CD4 SP thymocytes and strengthens its signaling. These factors may affect the emergence and replication of CXCR4-tropic over CCR5-tropic viruses [62]. The CXCR4-tropic HIV strain LAI is more efficient in infecting immature than mature thymocytes [48]. HIV coreceptor switch from CCR5 to CXCR4 usage has been linked to accelerated disease progression. In this regard, phylodynamic analysis has suggested that the thymus is an important site where HIV variants that use different coreceptors may be generated and amplified [63]. 

CXCR4 WHIM mutations may reduce thymic output, which may contribute to the T lymphopenia and restricted T-cell repertoire reported in WHIM patients [3]. The thymus has not been examined directly in any WHIM patients; however, in the WHIM mouse model, the thymus has been reported to have a 30% reduction in cellularity. On the other hand, thymus weight, architecture, and frequency and spatial distribution of thymocytes are all normal in WHIM mice [26]. One of the contributing factors may be the *Cxcr4^+/1013^* WHIM allele-dependent reduction in early thymic progenitors (Lin^−^CD4^−^CD8^−^c-Kit^+^CD44^high^CD25^−^) and DN1-4 cells, resulting in a reduction of circulating naïve T cell (CD3^+^CD44^−/low^CD62L^high^) numbers [33]. DP and SP CD4 thymocytes from WHIM mice display increased chemotaxis towards CXCL12 and reduced subsequent internalization of CXCR4 [26]. Enhanced CXCR4-dependent T cell migration capacity is likely to affect selection, maturation, and thymic output, all of which warrant further investigation.

### 2.3. Secondary Lymphoid Organs

In WHIM mice, splenic architecture is normal, but both the size and number of lymphoid follicles are markedly decreased [26], associated with a 45% reduction in cellularity which involves all major splenocyte subsets. Of note, the least affected cell populations are effector/memory CD4^+^ and CD8^+^ T cells. In contrast, the axillary and inguinal lymph nodes display increased cellularity in these mice, especially for the naïve CD4^+^ and CD8^+^ T cell subsets which may also contribute to the CD3^+^ lymphopenia in peripheral blood. In addition, B and T cell zones are disorganized in WHIM mouse lymph nodes. Enhanced extramedullary hematopoiesis is also observed in WHIM mice. In the peritoneal cavity, the B cell compartment is defective, with an expanded B1a subset, which is derived from fetal liver precursor cells, and contracted bone marrow-derived B2 and B1b subsets [26]. Additionally, extramedullary hematopoiesis in the spleens of WHIM mice is associated with enhanced amounts of splenic CXCL12 and increased circulation of hematopoietic stem and progenitor cells from the blood to the spleen [33]. B220^+^/CD43^+^ pro-B cells are reduced in fetal liver from E18.5 *Cxcr4*-deficient embryos [64]. Furthermore, the in vitro culture of wild-type fetal liver cells in the presence of IL-7 results in the generation of a large proportion of pro-B cells, whereas the culture of *Cxcr4*-deficient fetal liver cells yields fewer pro-B cells [32]. It should be noted that neither spleen nor lymph node appear to serve as sources of lymphocytes recruited to the blood by AMD3100. On the contrary, splenectomy enhances the effects of AMD3100 in wild-type mice. In addition, AMD3100 increases lymphocyte numbers, especially CD8^+^ T cells and B cells in lymph node [65]. 

## 3. Lymphocytes

### 3.1. B Cells

On B cells, CXCR4 colocalizes and appears to function with the IgD-B-cell receptor (BCR). In particular, CXCL12 stimulation of B cells potentiates both IgD-BCR-dependent actin remodeling and PI3K/AKT and ERK signaling [66]. Conversely, B cells lacking IgD show reduced chemotaxis in response to CXCL12 [66]. In agreement with this, B cells from WHIM syndrome patients and WHIM knockin mice are more activated and susceptible to apoptosis in unchallenged mice. Likewise, CXCL12 stimulation upregulates the activation marker CD69 in B cells, and this response is aberrantly enhanced in cells from WHIM patients and WHIM mice which might impair B cell survival and long-term function, a form of activation-induced cell death. Consistent with this idea, one study has shown that WHIM B cell dysfunction can be ameliorated by simply reducing the immunogen dose, resulting in a prolonged Ig response [67]. 

In bone marrow, CXCL12-CXCR4 signaling in B cells induces B cell motility, and the gradual loss of CXCR4 results in reduced motility followed by egress of immature B cells from the bone marrow [68]. Consistent with this, the targeted deletion of *Cxcr4* in B cells causes premature migration of B cell precursors from bone marrow and their localization within splenic follicles. Consequently, mature B cells become reduced in the primary follicles and marginal zones of the spleen and aberrant B cell follicles form ectopically in intestinal lamina propria and Peyer’s patches. Functionally, T-independent antibody responses are dampened [69].

WHIM patients may have hypogammaglobulinemia, markedly reduced circulating B cells [1,3,4,15,17,18,19,35,36,38,43,70,71,72], restricted immunoglobulin heavy chain variable region diversity, impaired class switching [71], and poor or unsustained responses towards vaccines [1,3,4,19,20,38,71]. Hypogammaglobulinemia may involve one or more of the immunoglobulin classes. The incidence and severity of hypogammaglobulinemia varies widely among WHIM patients, with some patients having normal levels of Ig, and others having borderline or below-normal levels [1,4,5,17,18,20,37,71]. In contrast, hypogammaglobulinemia has not been reported in WHIM mice despite the fact that WHIM mice like almost all WHIM patients have severe B lymphopenia [26]. Moreover, the precise contribution of hypogammaglobulinemia to infection risk in WHIM patients is undefined [7], although anecdotal evidence suggests that patients who initiated Ig supplementation therapy early in life may have benefited by a reduction in the incidence of respiratory tract infections. In addition, patients who are not on prophylactic Ig therapy may be at higher risk of developing severe bronchiectasis. Badolato and Donadieu thus recommend early Ig replacement therapy to all WHIM syndrome patients, irrespective of whether they have hypogammaglobulinemia [20]. Although this is certainly a reasonable approach, it has not been formalized as a consensus standard of care or scrutinized in clinical trials.

Only a few careful studies of vaccine responses in WHIM patients have been conducted. The humoral response was studied in a 12-year old WHIM patient post vaccination with quadrivalent Gardasil^®^, an HPV vaccine comprised of virus-like particles for the low cancer risk HPV strains 6 and 11 and the high-risk strains 16 and 18. Although HPV-specific antibody titers were lower compared to healthy controls, the patient developed a response and her antisera could neutralize pseudovirions of HPV in vitro. Unfortunately, her humoral response was not monitored long term [70]. In a second study, reduced class switching was observed after immunization, which may be due to defective germinal center trafficking of lymphocytes [71] and defective organization of germinal center light and dark zones. These processes are regulated in part by CXCR4 and CXCR5 [73]. Additional studies are needed to define B cell responses to vaccines in larger numbers of WHIM patients and to test whether CXCR4 blockade and immunogen dose variation might be combined to elicit durable and protective responses in these patients, as suggested by studies in WHIM mice [67].

Since hypogammaglobulinemia has not been phenocopied in the WHIM mouse, it has not been possible to rigorously dissect the cellular and molecular mechanisms of this WHIM phenotype. In fact, IgG and IgM levels are elevated in the WHIM mouse, whereas IgA levels are comparable to levels found in wild-type mice [26]. Nevertheless, the inability to maintain Ig levels in the normal range in WHIM patients has recently been postulated to reflect defective differentiation and homing of antigen-specific plasma cells (PCs). In this regard, in the WHIM syndrome mouse model, irrespective of the immunization route, the antigen, or the adjuvant, immunization results in increased absolute numbers and proportion of antigen-specific functional PCs in the spleen and lymph node [74]. This has been associated with increased AKT phosphorylation upon CXCR4 signaling and BCR cross-linking [66,74]. Yet the antigen-specific PCs fail to localize properly and accumulate in the bone marrow, and serum titers of Ag-specific Abs are not sustained over time. The immature PCs accumulate in the bone marrow very early after immunization, whereas the antigen-specific PCs generated subsequently fail to home and localize [74]. 

It is of interest to note that the B cell is the leukocyte that is most responsive to AMD3100 in WHIM patients, increasing ~40-fold in the blood within several hours after drug administration [43]. The majority of B cells after mobilization display an immature phenotype (CD19^+^CD27^−^), and are specifically CD27^−^IgD^+^IgM^+^ naive (transitional) cells while some CD27^−^IgD^−^IgM^+^ immature B cells were also recruited [44]. In a subsequent phase 1 long-term clinical trial with low-dose AMD3100, in which all three WHIM patients responded well to the treatment and the incidence of infection and wart burden was reduced, the circulating B cells post mobilization were comprised mainly of naïve CD19^+^CD27^−^IgD^+^IgM^+^ cells [38]. The bone marrow serves as the likely source of AMD3100-recruited B cells [65]. Nevertheless, despite the increase in the cellularity after AMD3100 treatment and B cells demonstrating class switching in vitro, serum Ig levels could not be successfully elevated, and pneumococcal and diphtheria/tetanus vaccinations were not successful in eliciting antibody responses [38]. Hematopoietic stem cell transplantation (HSCT) has been reported to correct leukopenia including B lymphopenia and hypogammaglobulinemia in WHIM patients and has been successfully accomplished in three patients [35,72,75]. Of note, in one patient, although B cell numbers and IgG levels were increased, IgA levels remained low [35].

### 3.2. T Cells

Apart from HPV, WHIM patients do not typically acquire the types of infections seen in patients with severe T cell immunodeficiency [7]. Consistent with this, while T cell distribution and function are abnormal in WHIM syndrome, severe T lymphopenia and severe functional defects are not observed. As with B cells, CXCR4 signaling has been reported to costimulate TCR activation in human CD4^+^ T cells [76,77]. Upon CXCL12 stimulation, CXCR4 physically associates with the TCR and enhances ZAP-70 (protein tyrosine kinase zeta-associated protein) binding to the ITAM (immunoreceptor tyrosine-based activation motif) domain of the TCR. This results in prolonged activation of the mitogen-activated protein kinase, ERK, increased intracellular calcium ion flux, and increased activity of the transcription factor AP-1 [78]. In addition, CXCR4 costimulation induces F-actin polymerization, which enhances the number and stability of microclusters formed by the adaptor molecule SLP-76. Activated ZAP-70 phosphorylates two tyrosine residues on SLP-76 [79], resulting in enhanced proliferation [80] and upregulation of T cell activation molecules, including CD69 and CD25 [76]. Costimulation results in enhanced production of the cytokines Interferon-γ (IFN-γ), IL-10, and IL-4 [76,78]; conflicting results, however, have been reported for IL-2 [81]. CCR5 has also been reported to be recruited to the immunological synapse where it may physically interact with CXCR4 and provide costimulatory signals during anti-CD3-mediated T cell activation [81]. Reciprocally, TCR activation by anti-CD3 does not induce downregulation of CXCR4 on T cells, and accordingly enhances CXCL12-induced chemotaxis [76]. CXCL12-mediated chemotaxis is mediated by PI3K activation resulting in the accumulation of phosphatidylinositol-(3,4,5)-trisphosphate (PIP_3_) [82] and the activation of both ZAP-70 [77,83] and the docking protein Dok-1, also known as RasGAP-associated p62 protein [84]. 

Since CXCR4 costimulates TCR activation, it might be anticipated that gain-of-function WHIM variants of CXCR4 would have augmented costimulatory activity. On the contrary, T cells from human WHIM patients are unable to form long-lasting immunologic synapses with antigen-presenting cells [85]. This may contribute to delayed IgG class switching [71]. Along with a weakened immunological synapse, inhibitory mechanisms may also operate to hinder CXCR4-mediated costimulation of T cells. Nonetheless, WHIM patient lymphocyte proliferation in response to phytohemagglutinin is normal [4] and T cells from WHIM patients are intrinsically capable of activation by anti-CD3 and anti-CD28 stimulation [85]. 

Compared with healthy controls, CXCL12 less effectively downregulates cell surface CXCR4 on T cells from WHIM patients [4,6]. Accordingly, WHIM T cells display enhanced chemotactic responses to CXCL12 [3,4,86]. In WHIM patients, total circulating levels of CD4^+^ T cells may be either normal or only slightly reduced, whereas levels of CD8^+^ T cells may be more affected [3,17,18,35,36,38,87]. More detailed analysis has revealed that circulating levels of naïve subsets of CD4^+^ and CD8^+^ T cells are frequently reduced in WHIM patients, probably due to reduced thymic output as suggested by their restricted T-cell repertoire [3]. The levels of effector memory cells on the other hand may be in the normal range or even increased. 

The underlying mechanisms responsible for this pattern may include defects in homeostatic proliferation, which is regulated in part by CXCR4. However, CXCR4 does not appear to play a role in the self-renewal of CD8^+^ memory T cells upon antigen rechallenge [88]. Moreover, naïve CD4^+^ T cells home to bone marrow in wild-type mice, and AMD3100 appears to mobilize both naïve and memory T cells from bone marrow [65]. CXCR4 is also required for homing of naïve and memory CD8^+^ T cells to bone marrow [89]. AMD3100 is also effective in recruiting naïve, effector, memory, and regulatory T cells into the circulation of rhesus macaques [90]. In the phase 1 dose escalation study of AMD3100 in three unrelated WHIM patients, CD4^+^ and CD8^+^ T cells belonging primarily to the effector memory phenotype (CD45RO^+^CD62L^−^CCR7^−^) were mobilized to the blood [44]. In one report, G-CSF was also found to recruit regulatory T cells from the bone marrow in healthy humans; AMD3100 might also recruit these cells from the same reservoir [91]. 

### 3.3. Natural Killer (NK) Cells

CXCL12-CXCR4 signaling is also critical for NK cell development [92] and trafficking [93]. In adult mice conditionally deficient in CXCR4, NK cells are severely reduced in the bone marrow, spleen, and blood, and have decreased functionality [92]. NK cell egress from bone marrow is normally governed by the opposing balance of retention signals mediated by CXCR4 and egress signals mediated by sphingosine-1 phosphate receptor 5 [93]. Thus, in the WHIM mouse model, NK cells accumulate in bone marrow and lymph node, whereas their absolute numbers are reduced in spleen and blood [93]. In rhesus macaques [90] and wild-type mice [93], AMD3100 administration results in the mobilization of NK cells to the blood. In WHIM patients, circulating NK cell levels have been reported to be either in the normal range [18,35,71] or reduced [19,38,71]. Interestingly, neutrophils regulate terminal maturation and functionality of NK cells. Severe congenital neutropenia patients have a higher frequency of less mature NK cells (CD56^bright^) which are hyporesponsive to stimulation as compared to cells from healthy controls. It will therefore be interesting to examine the maturation state and effector functions of NK cells in bone marrow, blood, and spleen of WHIM mice and circulating NK cells in WHIM patients [94]. AMD3100 has been reported to normalize NK cell levels in WHIM patients where they were low [38]. In patient WHIM-09, although chromothripsis selectively deleted the WHIM allele from the myeloid lineage, NK cell levels in blood were normal, whereas both B and T cell levels were low [15]. The role of NK cells in recurrent infections and HPV pathogenesis in WHIM patients is still undefined and can serve as an active area of research in the future.

## 4. The Question of HPV Susceptibility

For unknown reasons, WHIM patients are exceedingly susceptible to HPV infection, so much so that many novel serotypes of the virus have been discovered in these patients [95]. Thus, CXCL12-CXCR4 signaling seems to be important for controlling HPV infection and/or pathogenesis, including HPV-positive squamous cell carcinoma. This has been studied experimentally in a transgenic mouse model of squamous cell cancer induced by the expression of the HPV16 early region oncogenes E6 and E7 driven by the K14 keratinocyte promoter. In this model, AMD3100 treatment reduced keratinocyte hyperproliferation, immune cell infiltration, and HPV-induced epidermal neoplasia [96]. 

CD8^+^ T cells are well-known to limit HPV pathogenesis and tumorigenesis [97,98,99]. Multiple factors may alone or in concert impede CD8^+^ T cell activity against the virus in WHIM patients. First, the circulating numbers of naïve T cells and total CD8^+^ T cells are often low [17,18,35,36,38,87]. Second, the TCR repertoire may be impaired [3]. Third, CXCR4 modulates T cell activation at many levels, all of which can be dysregulated by WHIM mutations. Another population relevant to HPV control is the plasmacytoid dendritic cell (pDC) which generates Type I IFN. Production of pDCs and trafficking of pDCs in tissues are both regulated by CXCR4, and in WHIM patients, pDCs have been reported to be significantly reduced in the circulation and incapable of secreting IFN-α upon Toll-like receptor-9 activation [100]. It is possible that the mechanism of wart regression in patient WHIM-09 involved numerical and functional correction of the pDC portion of her myeloid lineage, although this has not been studied directly [15]. Changes in the cells of the myeloid lineage may affect the functioning of lymphocytes. Therefore, detailed investigation of both myeloid and lymphoid compartments will be required to fully delineate the role of CXCR4 in the control of HPV infection. 

## 5. Therapeutic Considerations

**Figure 2:**
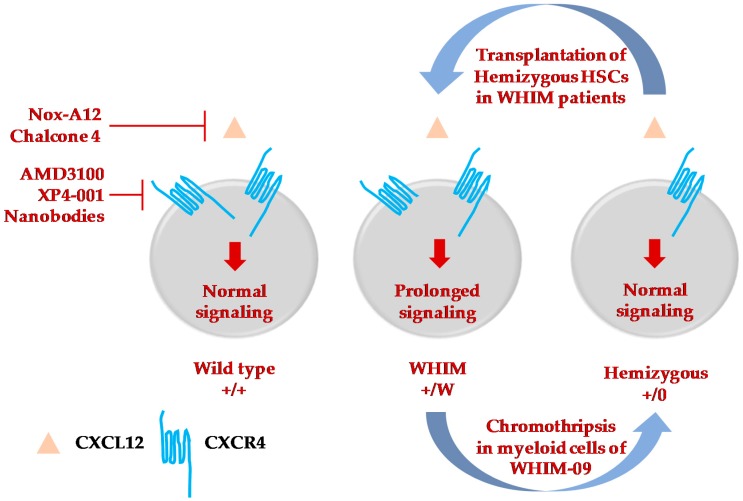
Therapeutic strategies to treat and cure WHIM syndrome. Selective inhibitors or molecules to specifically target either CXCL12 [102,103] or CXCR4 [38,43,44,101,104] have been developed and are at various phases of testing and clinical trials to mobilize immune cells from bone marrow of WHIM patients. Among the four patients to be cured of WHIM syndrome, three of them underwent successful hematopoietic stem cell transplantation [35,72,75]. The fourth patient, WHIM-09, was fortuitously cured by chromothripsis which occurred in a single HSC, which resulted in repopulation of myeloid but not lymphoid cells, cessation of recurrent bacterial infections and clearance of HPV-induced warts [15]. This suggested the possibility of inactivating the *CXCR4* WHIM allele by gene editing and transplantating autologous edited *CXCR4*-hemizygous HSCs as a possible cure for WHIM syndrome. Transplantation of *Cxcr4^+/0^* HSCs is successful in the reconstitution of blood cells in WHIM mice [34].

The natural history of WHIM syndrome is not defined. Moreover, current treatment approaches are non-specific, and their safety and efficacy have not been established through clinical trials [20]. Anecdotally, although intravenous Ig replacement and G-CSF treatment improve hypogammaglobulinemia and neutropenia and are thought to be clinically beneficial, some patients may still suffer recurrent infections and persistent warts resulting in substantial morbidity and in some cases premature mortality. Therefore, new, directed, and long-term therapies and cure strategies are needed, including potent and selective CXCL12/CXCR4 antagonists, HSCT, and gene editing (Figure 2). Although AMD3100/plerixafor is US Food and Drug Administration-approved for HSC mobilization and has shown promise in clinical trials in WHIM syndrome, it has poor bioavailability and possesses a short half-life in vivo, and therefore must be administered parenterally. The orally bioavailable CXCR4-selective antagonist, X4P-001 (X4-Pharma) has been reported to be well-tolerated and able to increase the ANC and ALC (which increased in greater proportion than the ANC) in a dose-dependent manner during a phase II trial consisting of four WHIM syndrome patients [101]. The oligonucleotide NOX-A12 is a CXCL12-specific inhibitor that has a longer half-life than AMD3100 and also mobilizes lymphocytes and other leukocyte subsets in healthy controls [102]. The small-molecule inhibitor Chalcone 4 is also specific to CXCL12 and inhibits CXCR4–CXCL12 interaction [103]. Recombinant single-domain anti-CXCR4 antibody fragments known as nanobodies have been developed that are potent inhibitors of *Cxcr4^+/1013^*-signaling [104]. These therapies require further investigation to assess their hematological effects as well as their safety and efficacy in generating vaccine responses and reducing HPV-induced wart burden and recurrent bacterial infection frequency during prolonged administration in WHIM patients.

To date, only four patients have been clinically cured of WHIM syndrome, of which three received allogeneic hematopoietic stem cell transplantation [35,72,75], whereas the fourth patient, WHIM-09, underwent spontaneous chromothripsis as mentioned previously [15]. Serendipitous removal of the disease allele from cells specifically belonging to the myeloid and not the lymphoid lineage cured patient WHIM-09 [15]. Thus, this quite remarkable experiment of nature is not informative for how WHIM mutations in lymphoid cells drive the clinical manifestations of the disease. HSCT was successful in restoring humoral immunity and peripheral blood counts without causing graft-versus-host disease (GVHD) [35,75]. In the third transplanted patient, mild acute GVHD (skin, grade I, stage I) was observed [72]. In addition to durable reconstitution of αβ T cells, allogeneic HSCT was reported in one WHIM patient to result in a transient increase in the proportion and number of γδ T cells, which the authors attributed to temporary hindrance of β-selection in the thymus post transplantation [72].

Gene editing-based gene therapy is also now under investigation as a cure strategy in WHIM syndrome, as in other inherited diseases of the blood. Our recent data suggest that WHIM allele deletion rather than correction may be a superior gene therapy strategy taking advantage of the apparent improved engraftment properties of *Cxcr4^+/o^* HSCs [34]. Moreover, the results imply that WHIM syndrome may be an excellent disease target for the development of gene editing as a cure approach in inherited diseases of the blood.

## Abbreviations

BCR	B-cell receptor
CLP	Common lymphoid progenitor
CMJ	Corticomedullary junction
CMP	Common myeloid progenitor
DN	Double negative
DP	Double positive
G-CSF	Granulocyte colony-stimulating factor
GVHD	Graft-versus-host disease
HSC	Hematopoietic stem cell
HSCT	Hematopoietic stem cell transplantation
Ig	Immunoglobulin
LN	Lymph node
NK	Natural killer
PCs	Plasma cells
pDCs	Plasmacytoid dendritic cells
SCN	Severe congenital neutropenia
SP	Single positive
TCR	T-cell receptor
WHIM	Warts, hypogammaglobulinemia, infections, and myelokathexis
